# The 100 Days Mission—2022 Global Pandemic Preparedness Summit

**DOI:** 10.3201/eid2903.221142

**Published:** 2023-03

**Authors:** Dimitrios Gouglas, Mario Christodoulou, Richard Hatchett

**Affiliations:** Coalition for Epidemic Preparedness Innovations, Oslo, Norway

**Keywords:** 100 Days Mission, pandemic, bioterrorism and preparedness, conference, 2022

On March 7 and 8, 2022, the Coalition for Epidemic Preparedness Innovations (CEPI) and the UK government cohosted the Global Pandemic Preparedness Summit in London, UK (*1*). More than 300 participants from governments, academia, industry, philanthropy, and civil society gathered to explore a proposed response to the next “Disease X” by making safe, effective vaccines, therapeutics, and diagnostics within 100 days of identification—a goal that the UK government designated the 100 Days Mission (*2*). This goal was originally articulated by CEPI, an innovative global partnership between public, private, philanthropic, and civil society organizations launched in Davos, Switzerland, in 2017 to develop vaccines to stop future epidemics. The 100 Days Mission concept is part of CEPI’s 2022–2026 pandemic plan and has been endorsed by the Group of Seven forum, the Group of Twenty forum, and other governments around the world. Several vaccine developers are exploring strategies to achieve this aim (*3*–*5*). Reflecting on the devastation caused by the COVID-19 pandemic, multiple summit speakers highlighted the crippling health, economic, and social impacts of the pandemic (*6*,*7*). The world’s continued vulnerability to such threats was a central theme of the summit.

## The 100 Days Mission

Speakers noted that delivering on the 100 Days Mission will require focus and sustained commitment to innovation, equity, and collaboration. Summit participants agreed that bringing together such an ambitious effort would take time, substantial resources, and political will, all necessary to combat the growing threat posed by pandemics.

CEPI has embraced the concept of studying so-called prototype pathogens as a critical element of preparedness (*8*). By developing vaccines on rapid-response platforms against exemplars of a given viral genus or family, researchers can address scientific challenges characteristic of that family in advance, providing an important head start on developing vaccines against related threats (*9*). Such prototype vaccines will need to be tested, at a minimum, for clinical safety and immunogenicity. Gathering such data and experience will build confidence in the approach and inform regulators as they make decisions about the emergency authorization of vaccines against related pathogens.

Speakers highlighted the need for tools and innovations that enable rapid research and development (R&D) and manufacturing responses, including the optimization of clinical trials. They also underscored the need for master protocols, enabling faster data sharing and balanced research participation between high-income countries and low-income and lower-to-middle-income countries, and innovations in clinical trial design and execution to compress timelines for delivering trial outcomes. Additional recommendations included standardization of animal models and assays to assess vaccine candidates and avoid duplication of efforts, as well as improved global coordination of surveillance and data sharing connected with R&D efforts. Speakers urged development of global regulatory paradigms, ones with new platforms and requirements of emergency response that would take account of accumulating experience but not compromise on safety or quality. Summit participants also noted that regulators must come to a consensus regarding requirements for clinical trial data, become more familiar with technology platforms through review of data across various pathogens over time, and provide guidance on the feasibility of alternative pathways to emergency use approval, especially in the context of the 100 Days Mission (*10*).

## Improving Equitable Access

A common theme throughout the summit was global disparity in access to life-saving biomedical products, particularly in the context of epidemic and pandemic diseases. Minimizing the time between the emergence of new threats and the availability of medical countermeasures for affected populations is paramount. Despite record-breaking development speeds, COVID-19 vaccines were not available quickly enough because of unprecedented supply-chain disruptions and gaps in manufacturing capacity and delivery systems across regions. Initiatives such as COVAX—the vaccines pillar of the Access to COVID-19 Tool (ACT) Accelerator, a global collaboration to accelerate development, production, and equitable access to COVID-19 tests, treatments, and vaccines—were set up to address this situation. However, such initiatives have lacked the financial resources to secure sufficient doses early through advance purchase agreements. Lack of solidarity and prevalence of vaccine nationalism rendered COVAX supply-constrained through much of 2021 (*11*).

## Boosting Vaccine Manufacturing

Political and industry participants at the summit explored the topic of boosting global manufacturing. Discussions acknowledged that a commitment to equity will require sustained investment in distributed manufacturing capabilities across regions. Speakers shared reports of progress being made on several fronts: the World Health Organization (WHO) and partners are setting up mRNA vaccine technology transfer hubs in South America (*12*) and South Africa (*13*); Moderna (https://www.modernatx.com) plans to set up an mRNA manufacturing facility in Kenya (*14*); SK bioscience (https://www.skbioscience.com) plans to produce routine vaccines in preparation for Disease X that can quickly transfer to large-scale manufacturing if a new pandemic occurs; and WHO has designated South Korea as a biomanufacturing training hub (*15*). Those speakers noted, however, that such efforts and capabilities cannot be sustained unless manufacturing remains functional and commercially viable outside pandemics.

## Global Pandemic Preparedness Needs Global Collaboration

There was consensus among speakers that responding quickly and effectively to pandemic threats requires close collaboration, leveraging the individual and collective strengths of the public and private sectors, and the normative function of multilateral organizations, such as WHO. WHO member states are working to establish the rules of the road for future epidemics and pandemics, including equitable sharing of countermeasures (*16*).

## Concluding Thoughts

The summit concluded with representatives from industry, philanthropy, and governments pledging their political and financial support of nearly $1.6 billion against a target of $3.5 billion for CEPI’s 5-year pandemic plan and 100 Days Mission (*17*). In doing so, supporters emphasized the need to maintain momentum and break the cycle of panic and neglect by supporting rapid response technologies, including the testing of such technologies for clinical safety and immunogenicity against prototype pathogens to build confidence in accelerated R&D responses to outbreaks. More tools and innovations that enable rapid R&D and manufacturing responses will, in turn, be needed, including standardized animal models and assays, accelerated trial designs, improved rapid diagnostics, laboratory and clinical trial networks that enable data sharing, and distributed manufacturing across regions.
